# Identification of m7G-Related LncRNA Signature for Predicting Prognosis and Evaluating Tumor Immune Infiltration in Pancreatic Adenocarcinoma

**DOI:** 10.3390/diagnostics13101697

**Published:** 2023-05-11

**Authors:** Jiawei Lu, Pusheng Yang, Lanting Yu, Ni Xie, Ying Wu, Baiwen Li

**Affiliations:** 1 Department of Gastroenterology, Shanghai Key Laboratory of Pancreatic Diseases, Shanghai General Hospital, Shanghai Jiao Tong University School of Medicine, Shanghai 201620, China; 2 Department of Obstetrics and Gynecology, Shanghai Key Laboratory of Gynecology Oncology, Renji Hospital, School of Medicine, Shanghai Jiao Tong University, Shanghai 200127, China

**Keywords:** m7G, lncRNA, pancreatic adenocarcinoma, overall survival, prognostic signature, immune infiltration

## Abstract

N7-Methylguanosine (m7G) modification holds significant importance in regulating posttranscriptional gene expression in epigenetics. Long non-coding RNAs (lncRNAs) have been demonstrated to play a crucial role in cancer progression. m7G-related lncRNA may be involved in the progression of pancreatic cancer (PC), although the underlying mechanism of regulation remains obscure. We obtained RNA sequence transcriptome data and relevant clinical information from the TCGA and GTEx databases. Univariate and multivariate Cox proportional risk analyses were performed to build a twelve-m7G-associated lncRNA risk model with prognostic value. The model was verified using receiver operating characteristic curve analysis and Kaplan–Meier analysis. The expression level of m7G-related lncRNAs in vitro was validated. Knockdown of SNHG8 increased the proliferation and migration of PC cells. Differentially expressed genes between high- and low-risk groups were identified for gene set enrichment analysis, immune infiltration, and potential drug exploration. We conducted an m7G-related lncRNA predictive risk model for PC patients. The model had independent prognostic significance and offered an exact survival prediction. The research provided us with better knowledge of the regulation of tumor-infiltrating lymphocytes in PC. The m7G-related lncRNA risk model may serve as a precise prognostic tool and indicate prospective therapeutic targets for PC patients.

## 1. Introduction

Pancreatic cancer (PC) is one of the deadliest human diseases, ranking as the third leading cause of tumor-related mortality in America. The 5-year survival rate of PC is only 11% [1]. Surgical resection and adjuvant chemotherapy tend to be the main opportunities to improve the long-term prognosis of patients with pancreatic carcinoma. However, over 80% of patients present with unresectable or metastatic disease at diagnosis as a result of vague symptoms at the early stage of the tumor [2]. Over the past several decades, there have been improvements in diagnostic procedures and systemic treatments for advanced disease, which have contributed to some, although modest, progress in patient outcomes [3]. To have a clinically significant impact, new approaches to screening high-risk individuals to detect pancreatic cancer at earlier stages are urgently needed.

Epigenetic modifications, whether acquired or inherited, regulate gene expression at the transcriptional level without modifying the sequence of DNA. These modifications contribute to multiple pathological processes, including tumorigenesis [4]. Over 100 distinct forms of RNA modification have been reported to date. The methylation of various RNA species has emerged as a crucial regulator of transcript expression [5]. N6-methyladenosine (m6A) is the most prevalent and abundant epigenetic modification, primarily found within the RRACH motif and distributed in the 3′UTR sections near stop codons [6]. Additionally, two modifications that occur throughout eukaryotic mRNAs are N1-methyladenosine (m1A) and 5-methylcytosine (m5C) [7]. Moreover, N7-methylguanosine (m7G) is described as a methyltransferase that attaches a methyl group to the seventh N position of guanine (G) in mRNA [8]. As one of the most prevalent kinds of posttranscriptional base alteration, m7G modification exerts a broad influence on RNAs, including mRNA, rRNA, and tRNA. Furthermore, it plays a key role in several biological processes, such as mRNA translation and transcriptional elongation, and functions as a significant disease diagnostic marker [9]. Recent research has demonstrated that tumor oncogenesis and development are regulated by m7G modification. For instance, METTL1-controlled changes to tRNA m7G made HCC cells resistant to lenvatinib by making it easier for EGFR pathway genes to be translated. Moreover, METTL1 may be a viable resistance prediction marker and intervention target [10]. In addition, in hepatocellular carcinoma, METTL1-mediated modification of tRNA m7G boosts target mRNA translation with an increased frequency of m7G-related codons. Patients with HCC have a poor prognosis as a result of ectopic expression of METTL1 [11]. Recent bioinformatics research has shown that some cancers are caused by the dysregulation of m7G-related genes [12,13,14]. However, it remains uncertain whether m7G modification is associated with PC.

Long noncoding RNAs are generated by their respective genes and have a promoter structure and polyA tail identical to those of messenger RNA. [15]. During the process of differentiation, several lncRNAs are generated by distinct splicing mechanisms. Several studies have demonstrated that the epigenetic, transcriptional, and posttranscriptional levels of downstream genes can be modulated by lncRNAs. These include histone processing, gene silencing, nuclear transport, transcriptional interference, and regulation, which are directly associated with the onset of numerous human disorders [16,17]. Methylation-related genes may impact malignant tumor growth by changing the methylation level of lncRNAs [18,19,20]. Nevertheless, the role of m7G modification-related lncRNAs in the progression of PC remains uncertain. It is thus critical to find m7G-associated lncRNA biomarkers for the early identification and prognosis evaluation of PC.

Hence, based on PC patient data obtained from The Cancer Genome Atlas dataset (TCGA) and bioinformatic and statistical analysis, we created an m7G-related lncRNA prognostic signature (m7G-LPS) to reliably predict the survival probabilities of PC patients. This model was designed to evaluate the overall survival of PC patients with a unique prognostic model based on m7G. Next, we developed a nomogram to predict the OS of PC patients. Additionally, we investigated the correlation between the relationship and immunotherapy responses. Using the publicly accessible drug sensitivity database, we finally identified potential medicines that target this m7G-related lncRNA profile. In conclusion, our study suggests that the risk model may provide a potential predictive tool and indicate factors that crucially regulate the dispersion of PC immune cells.

## 2. Materials and Methods

### 2.1. Preparation of Data

RNA sequence data and clinical characteristics of PC patients were downloaded from the Cancer Genome Atlas (TCGA) database and the Genotype–Tissue Expression Project (GTEx). The dataset contained 167 normal pancreatic tissues from GTEx and 178 PC tissues from TCGA, along with 4 adjacent peritumoral tissues. The R package “sva” was employed to standardize gene expression profile data from several databases in bulk. Thirty-five m7G-related genes were identified from the gene set enrichment analysis (GSEA) website (http://www.gsea-msigdb.org/gsea/login.jsp, accessed on 1 September 2022) and the published literature [21]. Patients with a follow-up time of less than a month (30 days) were omitted to eliminate bias in the subsequent analysis. Patients who lacked full clinical data were excluded from the subsequent analysis.

### 2.2. Identification of Differentially Expressed m7G-Related lncRNAs in TCGA and GTEx

Gene probes of the expression matrix were annotated based on the lncRNA annotation dataset obtained from GENCODE (https://www.gencodegenes.org/, accessed on 1 September 2022). The R package “limma” was utilized to evaluate and filter the differentially expressed lncRNAs between the control group and the PC group. The criteria used for selection were |log2 fold change (FC)| > 1, and false discovery rate (FDR) < 0.05, using data from the TCGA and GTEx cohorts. As a result, 464 different lncRNAs expressed in pancreatic cancer were selected from 171 normal tissues and 178 tumor tissues. Then, we screened lncRNAs co-expressed with 35 m7G-related genes. With the combined matrices, we conducted a Pearson correlation analysis (|coefficients| > 0.3, and *p* < 0.001) between 35 m7G-related genes and differentially expressed lncRNAs. Consequently, 169 differentially expressed m7G-related lncRNAs were found between normal pancreas tissues and PC tissues.

### 2.3. Construction of the Prognostic Risk Model of m7G-Related lncRNAs

We conducted a univariate Cox analysis with a threshold of *p* < 0.05 to identify m7G-related lncRNAs with prognostic value. Then, multivariable Cox analysis was utilized to construct a risk score. The following equation was employed to calculate the risk score: risk score = coefficient (lncRNAi) × expression (lncRNAi), where coefficient (lncRNAi) represents the survival correlation regression coefficient of every lncRNA while expression (lncRNAi) represents each lncRNA’s expression.

### 2.4. Evaluation of the Risk Model of 12 m7G-Related lncRNAs as an Independent Prognostic Factor in PC

Patients with PC were separated into two groups based on the prognostic risk score’s median value. The whole genome, m7G-related coding genes, and m7G-related and risk model lncRNA expression profiles were analyzed by principal component analysis (PCA). The overall survival (OS) of PC patients was plotted using Kaplan-Meier survival curves. To determine if the diagnostic and prognostic value of the risk score was confounded by other clinical variables, univariate and multivariate Cox regression analyses were performed. Clinicopathological characteristics were assessed for their diagnostic and prognostic significance using receiver operating characteristic (ROC) curves.

### 2.5. Functional Enrichment Analysis

After samples were split into two groups based on the risk score, highly enriched pathways in distinct risk categories were identified with the use of GSEA. There were 1000 gene set permutations performed for each analysis. A normalized *p*-value of 0.05 and a false discovery rate of 0.25 were regarded as significant gene sets. To acquire multiple GSEA diagrams, the top seven functions enriched in the high-risk groups were visualized with an enrichment lot. DEGs were enriched and evaluated by using KEGG and GO with the clusterProfiler package. The results of the enrichment were then exhibited as bubble diagrams and barplots using the ggplot2 and enrichplot packages.

### 2.6. Analysis of the Immune Microenvironment

CIBERSORT is a common instrument for calculating the percentage of invasive immune cells by identifying marker gene expression. By integrating the transcriptome data of all PC patients with the expression of marker genes from 22 distinct kinds of immune cells, we utilized CIBERSORT to obtain the distribution scores of tumor-infiltrating lymph cells. To improve the accuracy of the deconvolution algorithm, the CIBERSORT *p*-value and root-mean-square error for each sample file were computed using 100 permutations of the default signature matrix. We next filtered and chose data from PC tissues for further analysis using the CIBERSORT value of *p* < 0.05. The immune cell distribution of PC samples was analyzed via the CIBERSORT method. To investigate the relationship between immune infiltrating cells and the risk score, immune cell infiltration data files were acquired from TIMER 2.0. These files were then analyzed using various R programs, including limma, ggplot2, scales, and ggtext.

### 2.7. Evaluation of Drug Sensitivity

With the pRRophetic R package, we evaluated the effectiveness of the patients’ therapy in different risk subgroups based on the half maximal inhibitory concentration (IC50).

### 2.8. Cell Culture and In Vitro Experiments

Two human pancreatic cancer cell lines (PANC-1 and MiaPaCa-2) and the human normal pancreatic ductal epithelial cell line HPDE6-C7 were cultured in DMEM (Gibco, New York, NY, USA) supplemented with 10% fetal bovine serum (Gibco, United States), 1% penicillin, and 1% streptomycin. The cell lines were maintained at 37 °C with 5% CO_2_. Total cellular RNA was extracted by TRIzol reagent (Accurate Biology, Shanghai, China) to assess the expression level of m7G-related lncRNAs. Reverse transcription was performed using HyperScript III RT SuperMix for qPCR with the gDNA Remover Kit (R202, EnzyArtisan, Shanghai, China). PCR was performed using Universal SYRB qPCR Mix (Q204, EnzyArtisan, Shanghai, China). GAPDH expression served as an internal control to calibrate the expression level of RNA. The relative expression levels were calculated via the 2^−ΔΔCT^ method. Each PCR was carried out three times, and the results were analyzed with GraphPad Prism (version 9.0.0). The primer sequences for PCR amplification are listed in Supplementary Table S1. Cell proliferation was detected by the Cell Counting Kit-8 assay (CCK-8) and EdU assay, while cell migration was detected by transwell and wound healing experiments.

### 2.9. Statistical Analysis

The expression levels of 35 m7G-related genes in 178 PC tissues and 171 normal pancreatic tissues were compared using a one-way analysis of variance. Cytoscape was used to establish and visualize the coexpression network of 12 prognostic m7G-related lncRNAs and mRNAs. The correlation between the expression of 12 m7G-related lncRNAs and clinicopathological factors was analyzed using the R package ggpubr. The OS time of each group was compared using the Kaplan-Meier technique. The ROC curve was utilized to evaluate the prognostic accuracy of the risk score model. A one-way ANOVA was used to analyze the results of RT-qPCR. *p* < 0.05 was considered to indicate statistical significance. R software (version 4.1.3) was utilized for bioinformatic analysis.

## 3. Results

### 3.1. Identification of Differentially Expressed m7G-Related LncRNAs in Patients with PC

**Figure 1:**
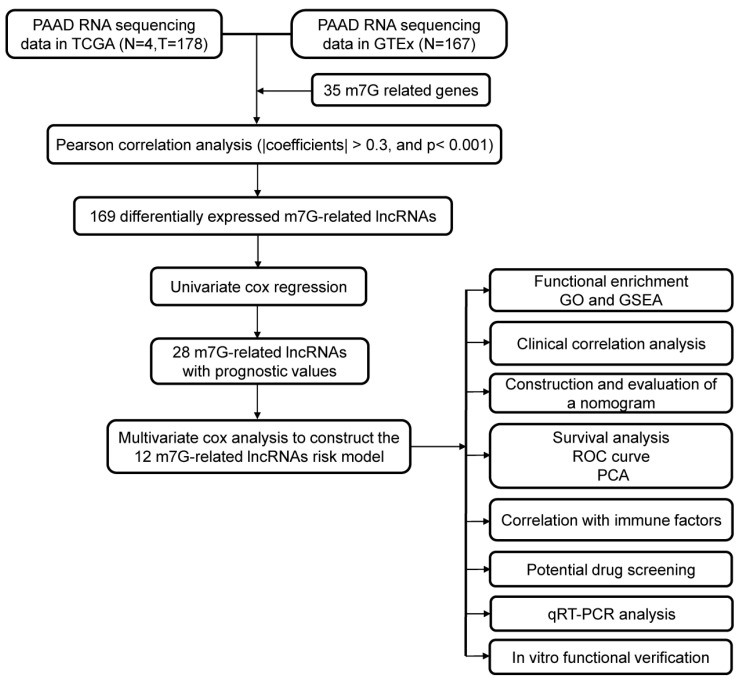
The flowchart of this study.

**Figure 2:**
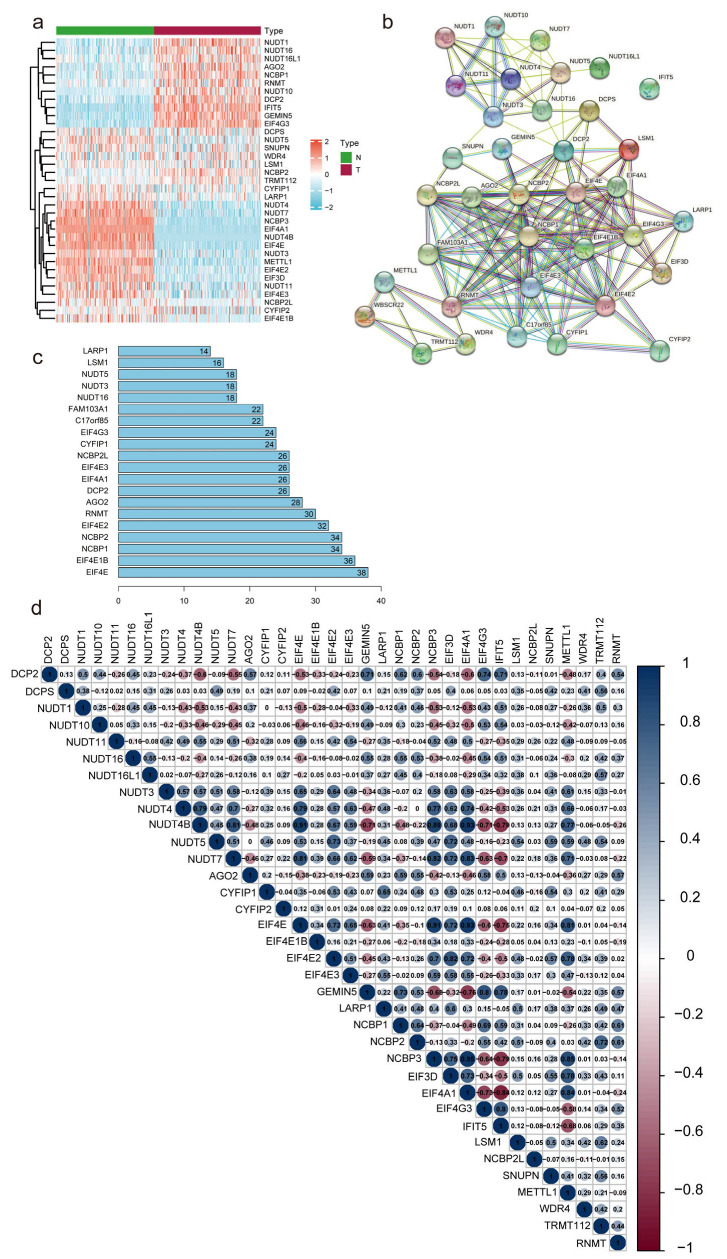
Correlations between m7G-related genes. (**a**) Heatmap depicting the differences in m7G-related genes expression between T and N groups. N, normal samples; T, tumor samples. (**b**,**c**) Network of protein–protein interactions illustrating the relationships between differentially expressed m7G-related genes. (**d**) Pearson correlation analysis of the m7G-related genes.

The process for risk model development and subsequent analysis is shown in Figure 1. We gathered data for 349 samples, including 178 tumor tissues and 171 normal tissues, from the TCGA and GTEx databases. Thirty-five m7G-related genes were selected from the GSEA database and previous literature. As shown in Figure 2a, the expression of m7G-related genes was considerably different between PC and normal tissues. Among m7G-related genes, the expression of DCP2, NUDT1, NUDT10, NUDT16, NUDT16L1, AGO2, GEMIN5, NCBP1, NCBP2, EIF4G3, IFIT5, TRMT112, and RNMT was notably elevated in the tissues of PDAC patients (Figure S1a, *p* < 0.01). We also noticed that the expression of NUDT11, NUDT3, NUDT4, EIF3D, EIF4A1, NUDT4B, NUDT5, NUDT7, NCBP2L, CYFIP2, CYFIP1, EIF4E3, EIF4E2, EIF4E1B, EIF4E, LARP1, NCBP3, SNUPN, and METTL1 was considerably decreased in tumor tissues compared to normal pancreas tissues (*p* < 0.01). However, no noticeable variations in the expression levels of DCPS, LSM1, and WDR4 between the groups. We then attempted to determine the associations among m7G-related genes. A PPI network of 35 m7G-related genes was constructed by STRING (Figure 2b). The number of nodes with more than 14 connections is depicted in Figure 2c. EIF4E, EIF4E1B, NCBP1, NCBP2, and EIF4E2 were defined as hub genes because their node degree values were 30 or more. EIF4A1 and NCBP3 had the strongest connections, with a coefficient of 0.95 (Figure 2d). Moreover, according to the total lncRNAs available in the TCGA and GETx databases, 464 different expressed lncRNAs in pancreatic cancer were selected with the criteria of |Log2FC| > 1 and *p* < 0.05 from 171 normal tissues and 178 tumor tissues. Then, based on Pearson correlation analysis (|coefficients| > 0.3 and *p* < 0.001) and the expression of 35 m7G-related genes, 169 differentially expressed m7G-related lncRNAs (|log2FC| > 1 and *p* < 0.05) were selected, among which 62 lncRNAs were downregulated and 107 lncRNAs were upregulated (Figure S1b).

### 3.2. Construction of the m7G-Related lncRNAs Risk Model

**Figure 3:**
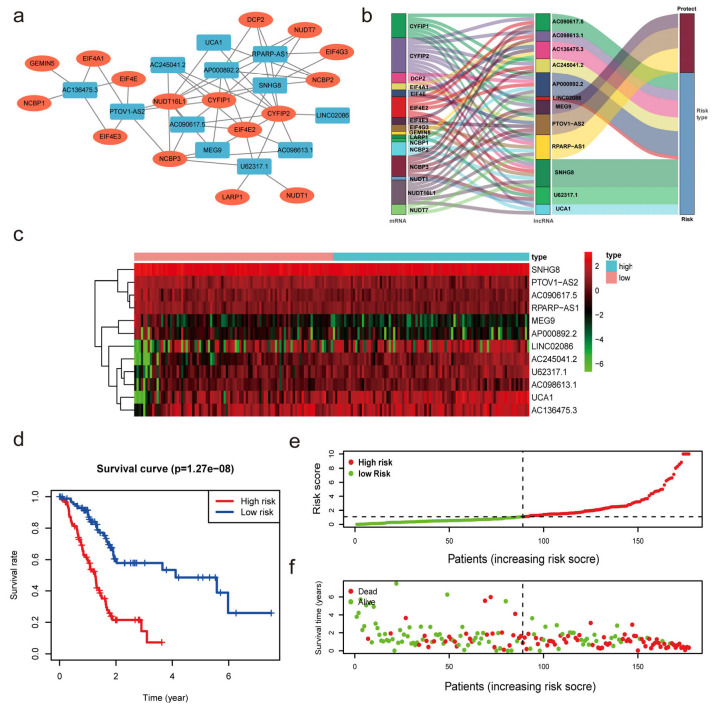
Construction of the m7G-related lncRNAs risk model. (**a**,**b**) Coexpression network of m7G-related lncRNAs−mRNAs. (**c**) Heatmap indicated the different expression of m7G-related lncRNAs in low- and high-risk groups. (**d**) Kaplan−Meier survival subgroup analysis for overall survival in high- and low-risk scores group (*p* < 0.001). (**e**,**f**) Risk score distribution based on the prognostic signature of m7G-related lncRNAs and survival status in patients with PC.

With univariate Cox regression analysis, we obtained 27 lncRNAs that had significant correlations with OS. In addition, we performed a multivariate Cox regression analysis. The results demonstrated that 12 of the 27 m7G-related lncRNAs had prognostic relevance. The following formula was employed to calculate the risk score: risk score = (0.61460602245472 × SNHG8) + (0.264591919818808 × U62317.1) + (−0.678414290153153 × MEG9) + (−0.816129541817646 × PTOV1-AS2) + (0.136027243890333 × LINC02086) + (0.71803727545072 × AC090617.5) + (0.229551930361753 × UCA1) + (0.314474780097137 × AC136475.3) + (−1.16089458841795 × RPARP-AS1) + (0.638671859460585 × AC245041.2) + (0.542733157457352 × AP000892.2) + (0.572444792342415 × AC098613.1). As demonstrated in Figure 3a, we developed a coexpression network to visualize 12 m7G-related lncRNAs-mRNAs in PC. The gene coexpressed with the most lncRNAs was CYFIP (*n* = 10), followed by CYFIP1 (*n* = 7) and NUDT16L1 (*n* = 7). SNHG8 (*n* = 8) was shown to be coexpressed with the highest number of mRNAs. The Sankey diagram exhibited the association between 35 m7G-related mRNAs and 12 risk lncRNAs, of which MEG9, PTOV1-AS2 and RPARP-AS1 were protective factors, whereas the rest were risk factors (Figure 3b).

The median value of the risk score was utilized to divide the PC patients into low- and high-risk subgroups to evaluate the validity and sensitivity of the predictive risk-related signature. The heatmap and violin plot demonstrated that all lncRNAs exhibited statistically significant variations between high- and low-risk pancreatic tissues. These findings suggest that m7G-related lncRNAs may be essential in PC progression (Figure 3c and Figure S1c). The Kaplan-Meier analysis of survival revealed that the high-risk group had a shorter survival duration than the low-risk group (*p* < 0.001, Figure 3d). This demonstrates that the risk score had prognostic significance. The association between the risk score and the related survival status was then depicted using risk curves and scatter plots (Figure 3e,f), which suggested that the mortality rate was positively correlated with the risk score. Thus, based on the 12 m7G-reated lncRNAs, we discovered m7G-related lncRNAs with significant prognostic value, and the predictive value of the m7G-LPS was established.

### 3.3. Correlation between Differently Expressed m7G-Related lncRNAs and Clinical Factors

**Figure 4:**
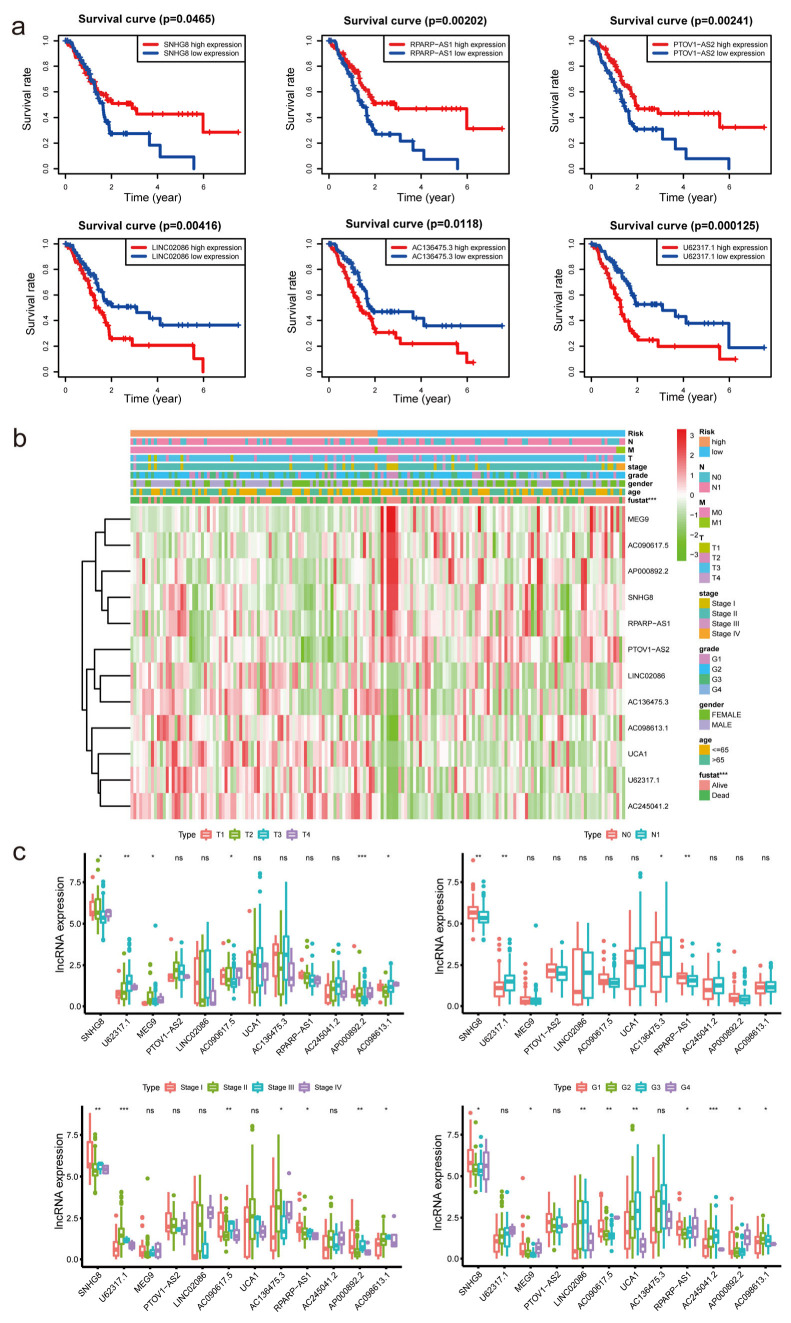
The association between the expression of m7G−LPS and clinicopathological factors. (**a**) Overall survival analysis of the 12 m7G-related lncRNAs between high- and low-risk groups. (**b**) Heatmap displaying the relationship between clinicopathological characteristics and different expressed m7G-related lncRNAs. (**c**) Box plot showing the different expression of 12 m7G-related lncRNAs in T, N, S, and G stage groups. * *p* < 0.5, ** *p* < 0.01, and *** *p* < 0.001. ns, no significant.

First, patients were separated into groups based on the expression of lncRNAs (high versus low). We noted considerable variation in the OS of the patients (Figure 4a and Figure S2a). The OS of individuals with high expression levels of AC098613.1, AC136475.3, AC245041.2, LINC02086, U62317.1, and UCA1 was inferior to that of individuals with low expression levels (*p* < 0.05). In contrast, patients with high expression levels of AC090617.5, AP000892.2, MEG9, PTOV1-AS2, RPARP-AS1, and SNHG8 had longer OS than those with low expression levels (*p* < 0.05). The heatmap demonstrated substantial differences (*p* < 0.001) in m7G-LPS survival status between groups with high and low risk. However, we failed to discover any appreciable variations by age, sex, pathological stage, grade, N stage, T stage, or M stage (Figure 4b). The link between clinicopathological characteristics and m7G-related lncRNAs was then thoroughly examined. We discovered that the expression of six lncRNAs differed significantly between patients grouped by T stage. It was discovered that four lncRNAs showed substantial differences between the two N-stage groups. Nine and seven lncRNAs were found to exhibit considerable differences in expression in the evaluation of patients grouped by grade and stage, respectively. Interestingly, we found that SNHG8 expression differed between all groups above, indicating that the gene may be the main predictive factor (Figure 4c). R0 resection rates were closely related to tumor metastasis and tumor prognosis. To further investigate the relation between R0 resection rates and m7G-LPS, we performed the Chi-square test. In Figure S3a, we conclude that R0 resection rates have a negative correlation with the expression of the m7G-LPS. The high-risk group had lower R0 resection rates than the low-risk group, which meant that the high-risk group had a higher probability of cancer metastasis. However, no significant difference was found between R0 resection rates and the expression of SNHG8 (Figure S3b). In 2020, Chan-Seng-Yue et al. [2] conducted whole-genome and transcriptome analyses on 314 specimens from pancreatic cancer patients. Researchers labeled “basal-like-A,” “basal-like-B,” “hybrid,” “classical-A” and “classical-B”’ in pancreatic cancer. Clinically, basal-like-A tumors are enriched in metastatic disease, whereas basal-like-B tumors are enriched in resectable disease. The m7G-LPS may serve as a predictor of tumor invasion and resection effects, which means the model can distinguish basal-like-A tumors from basal-like-B tumors to some extent.

### 3.4. Construction of the Nomogram and Verification of the Prognostic Model Built Using m7G Related lncRNAs

**Figure 5:**
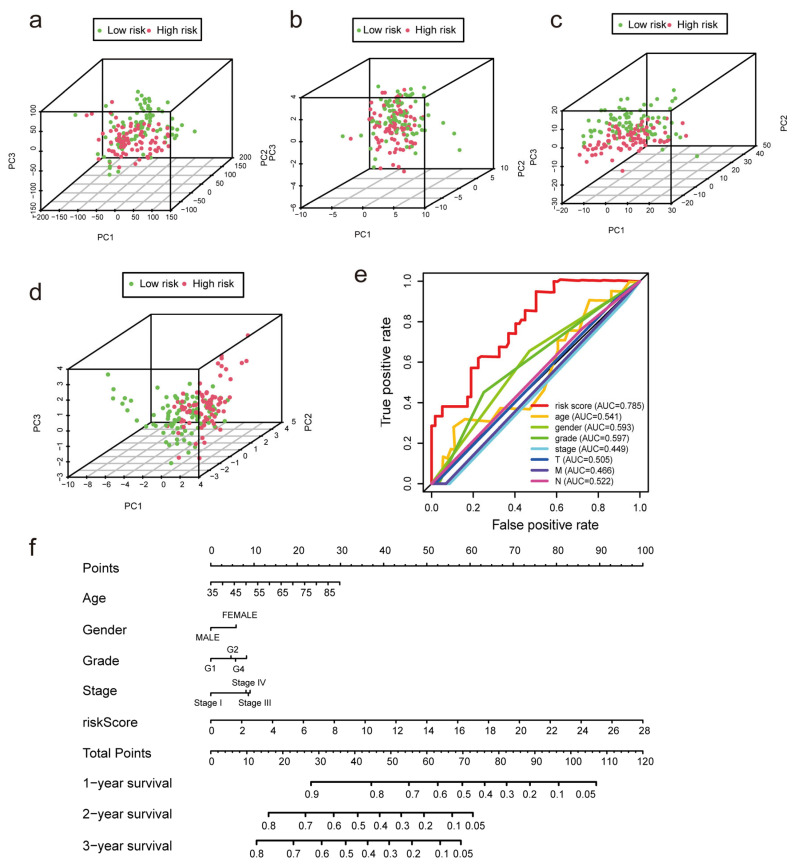
Verification of the model and establishment of a nomogram. (**a**–**d**) Principal component analysis was performed for the low- and high-risk groups based on the entire genes, m7G-related genes, m7G-related lncRNAs, and risk model lncRNAs. (**e**) ROC curves of the clinical characteristics and risk score. (**f**) The nomogram predicts the probability of overall survival at 1, 3, and 5 years.

We performed univariate and multivariate Cox regression analyses to determine whether m7G-LPS might be employed as an independent prognostic factor. The risk score was found to be a crucial predictive indicator that may be valuable independent of sex, age, grade, TNM stage, and pathological stage. The univariate Cox regression analysis showed that the risk score had an HR of 1.219 and a 95% confidence interval of 1.161–1.281 (*p* < 0.001). Moreover, in the multivariate Cox regression analysis, the HR of the risk score was 1.244, and the 95% confidence interval was 1.178–1.314 (*p* < 0.001) (Figure S2b). The distinct patterns of m7G distribution on the expression profile of all genes, the expression profile of m7G-related genes, the expression profile of m7G-related lncRNAs, and the expression profile of 12 risk lncRNAs between the two subgroups were identified using PCA (Figure 5a–d). The area under the ROC curve (AUC) value of the risk score was assessed to determine its sensitivity and specificity for forecasting the prognosis of PC patients. We discovered that the risk score’s AUC value was 0.785, and it was higher when compared with the results of other clinicopathological factors (Figure 5e). According to the aforementioned findings, m7G-LPS was a highly important independent predictive factor for PC individuals. The nomogram, which may be in clinical practice to forecast a patient’s prognosis, was created simultaneously using the risk score (Figure 5f). 

### 3.5. Functional Enrichment Analysis Based on the m7G-LPS

**Figure 6:**
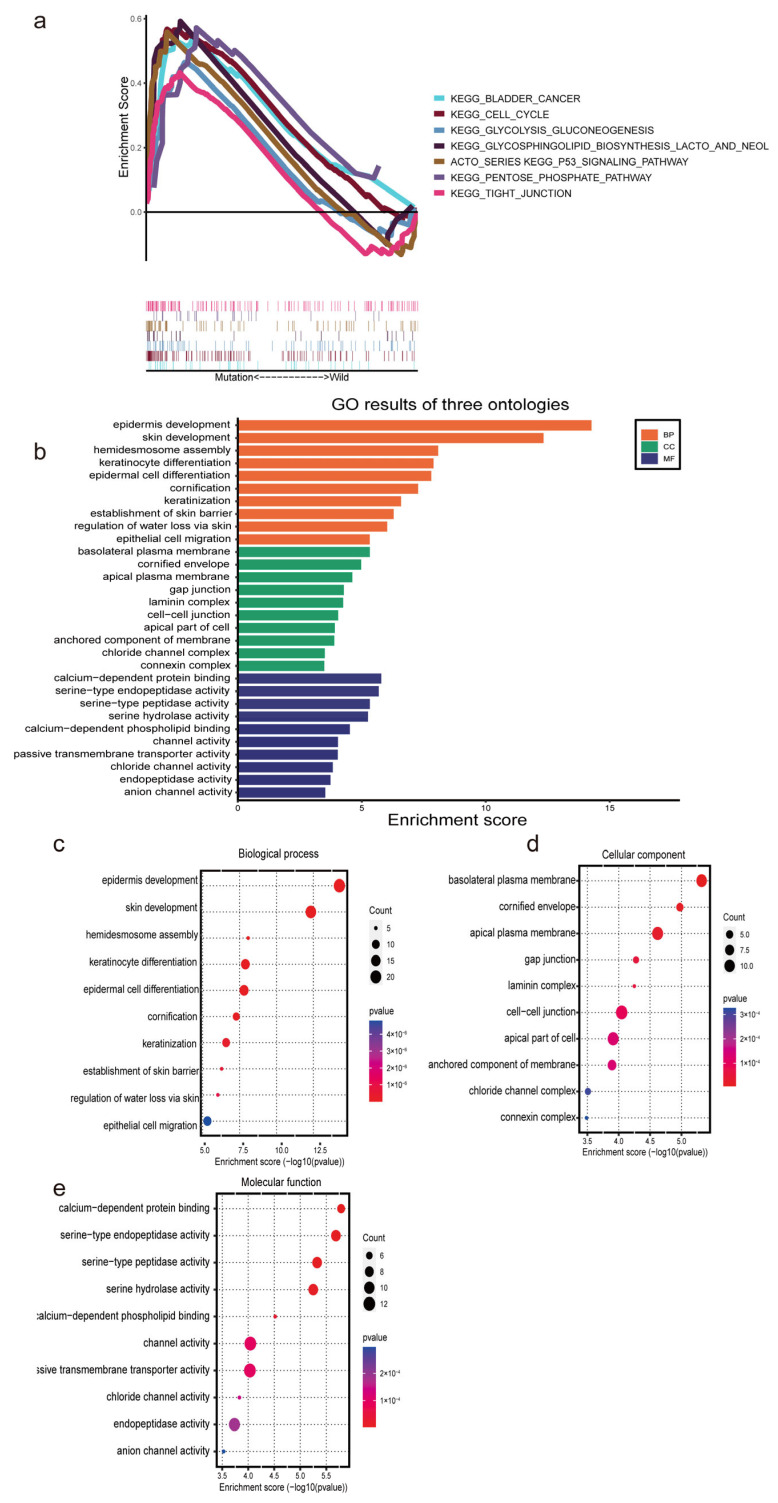
Functional enrichment analysis based on the m7G−LPS. (**a**) Gene set enrichment analysis shows seven significant enrichments of GO in the high-risk group. (**b**–**e**) The barplot and bubble chart illustrate the GO enrichment analysis among the differentially expressed genes.

Patients were split into high- and low-risk groups based on the m7G-LPS. We used GSEA to determine the abnormally enriched signaling pathways of m7G-related lncRNAs. Our analysis demonstrated that several tumor-associated pathways, including P53 signaling pathways, tight junction signaling pathways, and cell cycle signaling pathways, were associated with the high expression of m7G-related lncRNAs (Figure 6a). Additionally, the other enriched pathways of the high-risk group were strongly connected to glycolysis and included glycolysis, gluconeogenesis, glycosphingolipid biosynthesis, the lacto and neolacto series, and the pentose phosphate pathway. Hence, we speculate that m7G-related lncRNAs may participate in the development of pancreatic cancer through the glycolytic pathway. Gene Ontology functional enrichment analyses (Figure 6b) were used to annotate the functions of DEGs. As a result, some epidermis-related biological processes (BPs), such as epidermal development and epithelial cell migration, were identified. In addition, the cellular component (CC) findings revealed that the DEGs were mostly involved in the basolateral plasma membrane, gap junction, and cell-cell junction. The molecular function (MF) results revealed that the DEGs were involved in several serine-related processes, such as serine-type endopeptidase activity, serine-type peptidase activity, and serine hydrolase activity (Figure 6c–e). These findings present additional avenues to investigate the potential functions of lncRNAs associated with m7G in PC.

### 3.6. Estimation of Tumor-Infiltrating Lymph Cells Using the m7G-Related lncRNA Model

**Figure 7:**
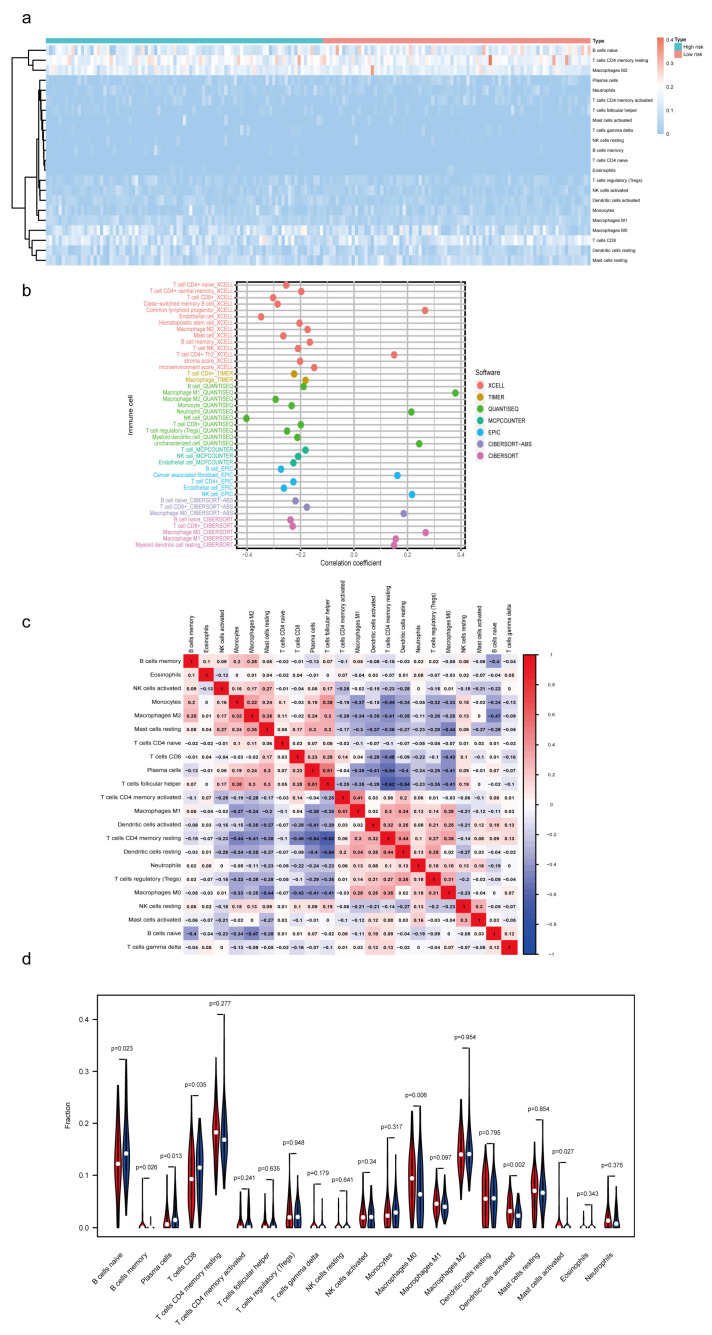
Immune landscape associated with the m7G-related lncRNA signature. (**a**) Heatmap of tumor−infiltrating immune cells in high- and low-risk groups. (**b**) Bubble graphic illustrating the correlation between risk score and immune cells. (**c**) Spearman correlation analysis of 21 tumor−infiltrating immune cells. (**d**) Violin plot showing the distribution of immune cells in high- and low-groups.

In this research, we evaluated the value of the tumor immune microenvironment in light of the risk factors associated with m7G-LPS. CD4 naïve T cells were not detected in the high- and low-risk groups. Hence, they were eliminated using the CIBERSORT screening tool (Figure 7a). Therefore, we investigated the relationship between the m7G-LPS score and the 21 immune cells that had infiltrated the tumor. We discovered that among them, memory B cells, M0 macrophages, activated dendritic cells, and activated mast cells were more enriched in the high-risk group than in the low-risk group. In contrast, the high-risk group had lower amounts of naive B cells, CD8 T cells, and plasma cells than the low-risk group (*p* < 0.05) (Figure 7d). In addition, we examined the relationships between 21 different kinds of tumor-infiltrating cells. We discovered that follicular helper T cells exhibited the highest correlation with resting memory CD4 T cells (r = −0.62), followed by activated memory CD4 plasma cells (r = −0.54) and resting dendritic cells and follicular helper T cells (r = −0.54, Figure 7c). The bubble graph illustrates the connection between immune cells and the risk score (Figure 7b). Most immune cells, especially NK cells and M2 macrophages of QUANTISEQ, endothelial cells, CD8+ T cells, and class-switched memory B cells of XCELL, had a negative correlation with the risk score. These results suggest that various tumor immune cell characteristics in PC patients could be distinguished based on the risk score of the m7G-LPS.

### 3.7. Clinical Application of the Risk Model

**Figure 8:**
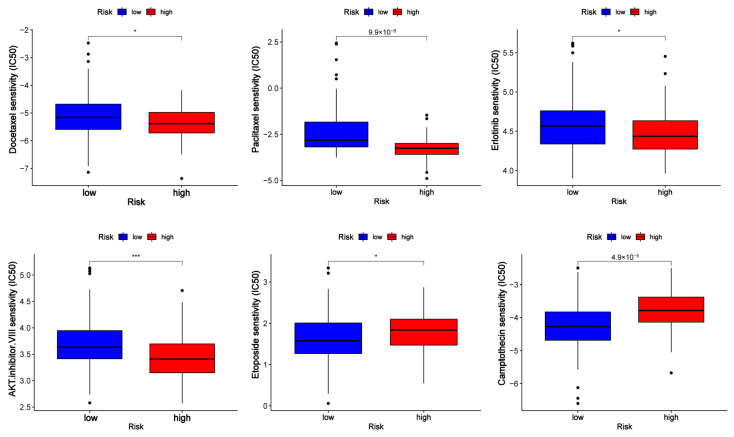
Half maximal inhibitory concentration (IC50) of drugs (docetaxel, paclitaxel, erlotinib, AKT inhibitor VIII, camptothecin, and etoposide) in low- and high-risk groups. * *p* < 0.5 and *** *p* < 0.001.

To evaluate the clinical applicability of the risk model, differences in drug sensitivity among different risk groups were investigated (Figure 8). As we can see from the figure, PC patients in different groups showed different drug treatment tendencies. The half maximal inhibitory concentration (IC50) was often used to test a drug’s ability to induce apoptosis. The sensitivity of tumor cells to the drug is inversely proportional to the IC50, with lower IC50 values indicating higher sensitivity. In view of the results, patients in high-risk categories responded favorably to docetaxel, paclitaxel, erlotinib, and AKT inhibitor VIII, while patients in the low-risk group were more sensitive to camptothecin and etoposide. According to GSEA and GO enrichment analysis, the cell cycle pathway was enriched in PC patients with high-risk scores. Some of the drugs that were effective in the high-risk group are typical antitumor drugs for pancreatic cancer, such as docetaxel and paclitaxel, whose antitumor mechanisms primarily target the cell cycle. Erlotinib is a targeted inhibitor of the epidermal growth factor receptor (EGFR) [22], which is crucial for cancer proliferation. Previous research has demonstrated the significant activation of the AKT signaling pathway in pancreatic cancer. This activation leads to alterations in cancer cell metabolism, increased cell cycle, and decreased apoptosis. These changes are closely associated with a poor prognosis in patients with pancreatic cancer [23,24,25]. The AKT inhibitor VIII was developed based on 2, 3-diphenylquinoxaline, which was discovered through a high-throughput screening effort to identify compounds capable of inhibiting all three AKT isoforms [26]. This inhibitor effectively decreased cell proliferation and increased apoptosis by translocation of phosphatidylserine (PS) and induction of cleaved caspase-9, caspase-3, and PARP [27]. Therefore, AKT inhibitor VIII may serve as a promising new anticancer drug for PC patients.

### 3.8. Verification of Expression Level in PC Cells

**Figure 9:**
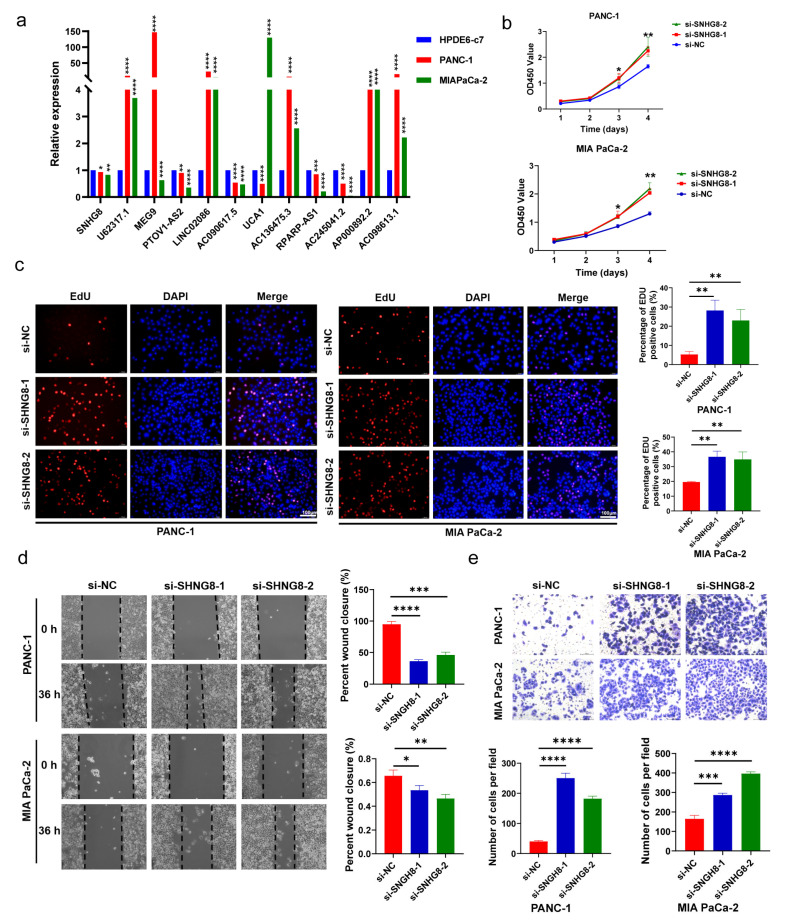
In vitro functional verification in PC cells. (**a**) Expression level of m7G-related lncRNAs in HPDE6-c7 cells and PC cells. (**b**,**c**) The proliferation ability of PC cell lines was detected by CCK-8 and EdU assays after SNHG8 knockdown. (**d**,**e**) Wound healing and Transwell assays were used to determine the migration capacities of PC cell lines after SNHG8 knockdown. * *p* < 0.5, ** *p* < 0.01, *** *p* < 0.001, and **** *p* < 0.0001.

In vitro qRT-PCR experiments were performed on one normal pancreatic ductal epithelial cell and two pancreatic cancer cell lines to assess the relative expression of lncRNAs (Figure 9a). The in vitro findings were not entirely in line with the TCGA data. In both PC cell lines, the expression of SNHG8, PTOV1-AS2, AC090617.5, and RPARP-AS1 was downregulated. Based on these results and the survival plots of patients categorized according to the expression of these factors, it was observed that high expression levels were associated with better survival, indicating that these factors may act as tumor suppressor genes. MEG9 was upregulated in PANC-1 cells and downregulated in MIAPaCa-2 cells, while UCA1 was downregulated in PANC-1 cells and upregulated in MIAPaCa-2 cells. For other lncRNAs, AC245041.2 was downregulated in both PC cell lines, and its low expression predicted worse survival. The expression of AP000892.2 was higher in both PC cell lines than in normal pancreatic duct cell lines. Interestingly, its high expression was associated with better survival outcomes, although the specific mechanism underlying this observation remains unclear and requires further investigation. Four lncRNAs (U62317.1, LINC02086, AC136475.3, and AC098613.1) were upregulated in both cell lines. Low expression levels of these lncRNAs were related to better survival, indicating that they function as oncogenes.

### 3.9. In Vitro Biological Function of SHNG8 in PC Cells

Combined with prognostic analysis and literature searches, we selected SNHG8 for further in vitro functional assays. We first silenced SNHG8 in PANC-1 and MIA PaCa-2 cell lines with siRNAs (Table S2). Then, the results of CCK-8 and EdU assays showed that the knockdown of SNHG8 significantly promoted the proliferation of PC cells (*p* < 0.05, Figure 9b,c). In addition, the migration rates were increased in Transwell and wound healing experiments (*p* < 0.05, Figure 9d,e) after SNHG8 was silenced in the two cell lines. Findings of CCK-8 assays showed that overexpression of SNHG8 significantly inhibited the proliferation of PC cells (*p* < 0.05, Figure S4a). Moreover, the migration rates decreased in Transwell and wound healing experiments (*p* < 0.05, Figure S4b,c) after SNHG8 was overexpressed in the two cell lines. Therefore, we concluded that SHNG8 functions as a suppressor gene in PC and might affect the prognosis of PC patients.

## 4. Discussion

Over the past several decades, pancreatic cancer has drastically increased in prevalence worldwide and is predicted to continue to be the main cause of cancer-related death. Although inherited genetic variables cannot be changed directly, they play a significant role in pancreatic cancer risk. In addition to shedding light on the etiology of pancreatic cancer, the identification of the genetic alterations that cause this disease offers the chance to direct early detection efforts [28].

RNA methylation, which comprises m5C, m1A, m6A, m7G, and other forms, is an essential epigenetic modification involved in posttranscriptional gene regulation. m7G is a prevalent modification that is involved in several physiological and pathological processes. Additional research is required to fully understand how m7G suppresses cancer, as has been shown to be the case [29]. In this work, we constructed a risk model of 12-m7G-related lncRNAs by downloading the gene expression profiles of 178 PC patients from the TCGA database. To the best of our knowledge, this is the first study to report the predictive evaluation of PC-associated lncRNAs connected to the m7G-related genes.

Noncoding RNAs (ncRNAs) account for most of the genome; they cannot code for proteins but generate noncoding transcripts that influence gene expression and protein activity. One of the two major classes of ncRNAs that have been well studied is lncRNA. LncRNAs are larger transcripts (more than 200 nucleotides in size) that are generated similarly to mRNAs but are not translated into proteins [30]. Despite being recently identified, lncRNAs have already been demonstrated to carry out cytoplasmic tasks such as miRNA sponging, modulation of protein activity, and posttranscriptional modification of particular mRNAs [31]. There is a vast variety of lncRNAs that have a wide range of functions, indicating that lncRNAs have the potential to behave as oncogenes and tumor suppressors. For instance, by decreasing NEAT1_1 expression in an m6A-YTHDF2-dependent way, methyltransferase-like 14 prevented renal cancer cells from proliferating and migrating [32]. The loss of XIST lncRNA impaired the differentiation of human mammary stem cells (MaSCs) and contributed to the emergence of highly malignant tumors [33]. LINC00467, which encodes the micropeptide ASAP (ATP synthase-associated peptide), promotes colorectal cancer progression by regulating the activity of ATP synthase directly [34]. However, the involvement of m7G-related lncRNAs in PC has not been published. Thus, we concentrated on the lncRNAs that were coexpressed with m7G-related genes in PC and created a predictive risk model for PC using bioinformatic and statistical techniques.

In our study, we identified differentially expressed m7G-related lncRNAs between pancreatic cancer tissues and normal tissues and uncovered the prognostic value of m7G-related lncRNAs in PC. More notably, a novel prognostic signature was identified and confirmed based on the differential expression of m7G-lncRNAs with prognostic value. By means of multivariate Cox and risk scoring methods, we constructed an m7G-LPS that divided all 178 PC patients into high- and low-risk groups with a significant difference in OS. According to Kaplan-Meier survival analysis, the high-risk subgroup showed worse OS compared with the low-risk subgroup, regardless of clinical features. The predictive accuracy of the m7G-LPS was validated by ROC curves for survival. Compared with traditional indicators such as cancer grade, stage, and age, the risk score performed better in terms of predicting the patient’s survival rate. In recent studies, a single molecular biomarker was the primary focus of the PC predictive risk model. 

However, diagnosis based on a single biomarker may be unreliable in the clinic due to individual differences, which may result in a large number of false–positive or false–negative results [35]. Moreover, it has been reported that circulating lncRNAs, a kind of cell-free nucleic acid (cfNA), could be proposed as a new type of potential biomarker for cancer diagnosis [36]. In particular, Arita et al. [37] demonstrated that plasmatic lncRNAs are resistant to degradation brought on by repeated freeze-thaw cycles and extended exposure to 45 °C and ambient temperatures. Hence, our research focused on 12 m7G-related lncRNAs, and this is the first study to describe a PC risk score model based on prognostic lncRNAs. In this research, 12 m7G-related lncRNAs were screened out, and 7 of them have never been reported in previous studies in PC (U62317.1, LINC02086, AC090617.5, AC136475.3, RPARP-AS1, AP000892.2, AC098613.1).

Furthermore, lncRNAs were reported to play pivotal roles in immune crosstalk between tumor cells and immune stromal cells in the tumor-immune microenvironment [38]. In this work, we carefully evaluated the relationship between m7G-related lncRNAs and the distribution of tumor-infiltrating immune cells. The results indicated quite a difference between the two subgroups, and the risk factors for m7G-LPS may be able to distinguish various tumor-infiltrating immune cell characteristics in PC. Therefore, this is the first study to discuss the correlation between m7G-related lncRNAs in PC and tumor-infiltrating lymph cells.

In vitro experiments were performed to evaluate the expression of lncRNAs in PC cells. According to the bioinformatic analysis, SNHG8 was differentially expressed in pathological stages, grades, N stages, and T stages. Thus, SNHG8 was selected for further study. It was silenced in two PC cell lines, and both proliferation and migration experiments proved that SNHG8 could inhibit the biological function of pancreatic cancer cells. The results indicated that SNHG8 acted as a suppressor gene in PC.

Despite our efforts to confirm the risk model’s stability from multiple angles, limitations still exist in our research. The transcriptome expression and clinical information of PC patients were downloaded from the TCGA and GTEx databases. Moreover, the expression of m7G-related lncRNAs was detected only in vitro without validation in in vivo experiments or PC patient tumor tissue. In addition, our study lacks experiments on molecular mechanisms. It is a preliminary step to identifying m7G-related lncRNAs that could affect pancreatic cancer patient survival. In our upcoming research, much work needs to be conducted to explore the mechanism by which these lncRNAs regulate PC progression.

## 5. Conclusions

We constructed a 12-m7G-related lncRNA prognostic risk model for PC patients, which was shown to have independent prognostic significance and offered an accurate survival prediction. Our research also expanded our knowledge of the regulation of tumor-infiltrating lymphocytes in PC. In conclusion, the m7G-related lncRNA risk model may indicate PC biomarkers or treatment targets.

## Data Availability

Publicly available datasets were analyzed in this study. These data can be found here: https://www.cancer.gov/, accessed on 1 September 2022 (TCGA).

## Supplementary Materials

The following supporting information can be downloaded at: https://www.mdpi.com/article/10.3390/diagnostics13101697/s1, Figure S1: Differentially expressed m7G-related genes between PC and normal tissues; Figure S2: Prognostic value of m7G-related lncRNA risk model; Figure S3: Correlation between R0 resection rates and the expression of m7G-LPS and SNHG8; Figure S4: In vitro functional verification in PC cells; Table S1: The primer sequences involved in the research; Table S2: The sequences of siRNA.
