# Supplementary material for: Identification of m7G-Related LncRNA Signature for Predicting Prognosis and Evaluating Tumor Immune Infiltration in Pancreatic Adenocarcinoma
Source: Diagnostics (Basel). 2023 May 11;13(10):1697. doi: 10.3390/diagnostics13101697 (PMC10217316; doi:10.3390/diagnostics13101697)
Supplement: Supplementary file 1 [file diagnostics-13-01697-s001.zip › supplementary files/legend.pdf]

Figure S1. Differentially expressed m7G-related genes between PC and normal tissues. (a) Different expressions of 35 m7G-related genes between PC tissues and normal tissues. (b) Volcanic map of m7G-related DEGs between PC and control samples with $\log_2$ FC >1 and $p < 0.05$. (c) Violin plot of 12 risk lncRNAs in low- and high-risk groups.

Figure S2. Prognostic value of m7G-related lncRNA risk model. (a) Survival analysis of 12 m7G-related lncRNAs between high- and low-risk groups. (b) Univariate and multivariate analysis of the clinical features and risk scores

Figure S3. Correlation between R0 resection rates and the expression of m7G-LPS and SNHG8. (a) Correlation between R0 resection rates and the expression of m7G-LPS. (b) Correlation between R0 resection rates and the expression of SNHG8.

Figure S4. In vitro functional verification in PC cells. (a) The proliferation ability of PC cell lines was detected by CCK-8 assays after overexpressing SNHG8. (b-c) Wound healing and Transwell assays were used to determine the migration capacities of PC cell lines after SNHG8 overexpressed. *$p < 0.5$, **$p < 0.01$, ***$p < 0.001$, and **** $p < 0.0001$. ns, no significant.
