# Supplementary figures and images for: Identification of m7G-Related LncRNA Signature for Predicting Prognosis and Evaluating Tumor Immune Infiltration in Pancreatic Adenocarcinoma
Source: Diagnostics (Basel). 2023 May 11;13(10):1697. doi: 10.3390/diagnostics13101697 (PMC10217316; doi:10.3390/diagnostics13101697)

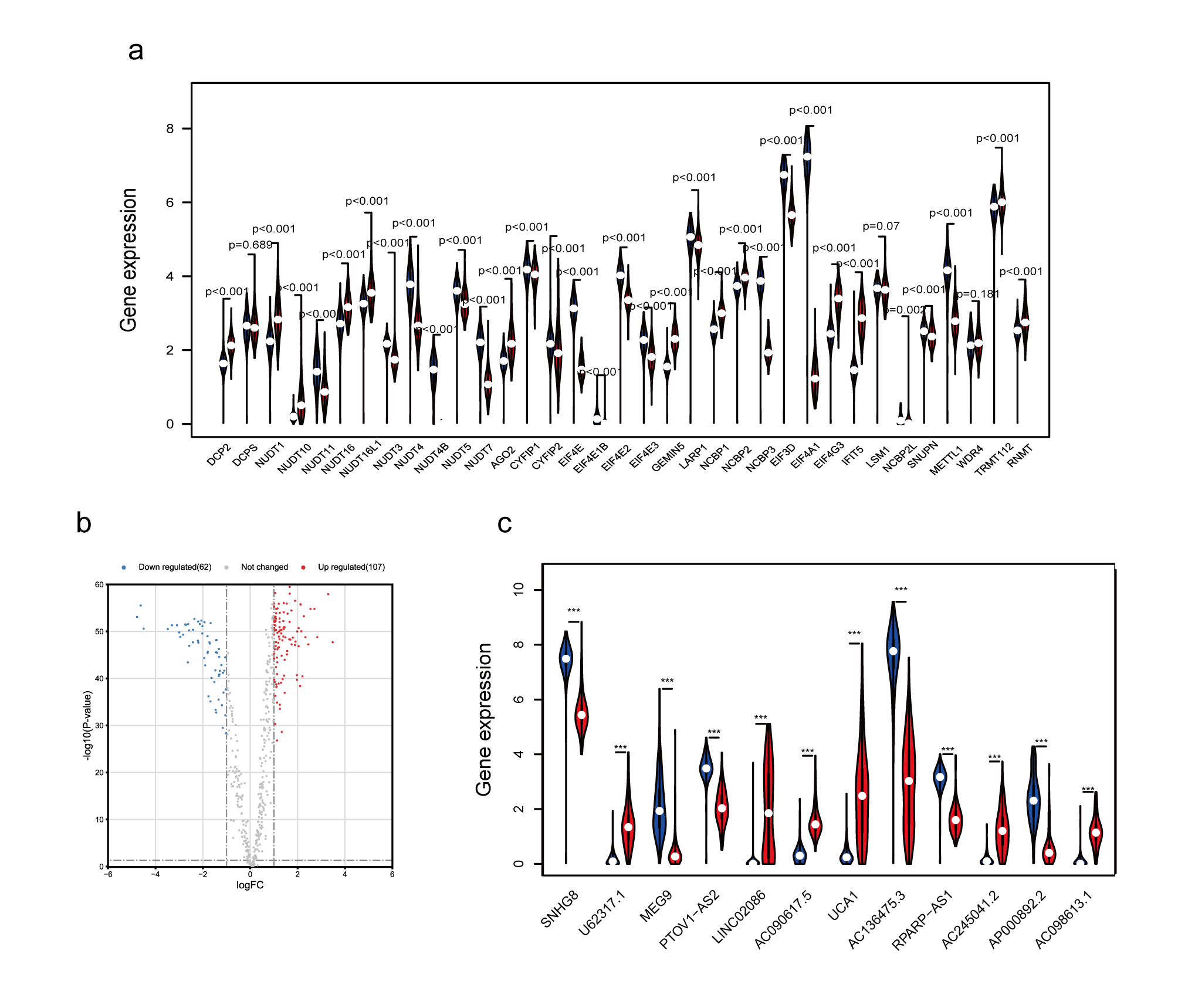

Supplement: Supplementary file 1 [file diagnostics-13-01697-s001.zip › supplementary files/Figure S1.tif]

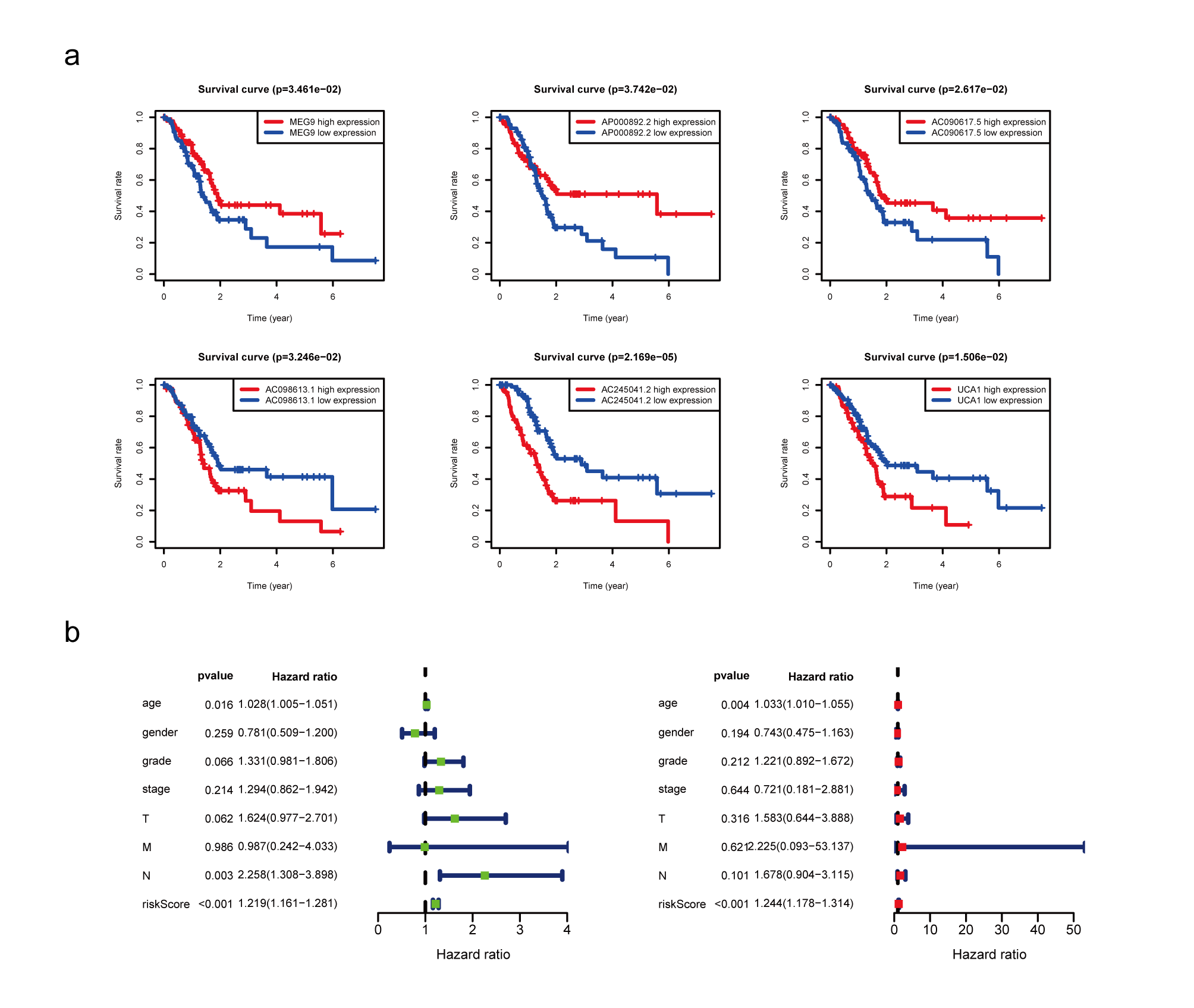

Supplement: Supplementary file 1 [file diagnostics-13-01697-s001.zip › supplementary files/Figure S2.tif]

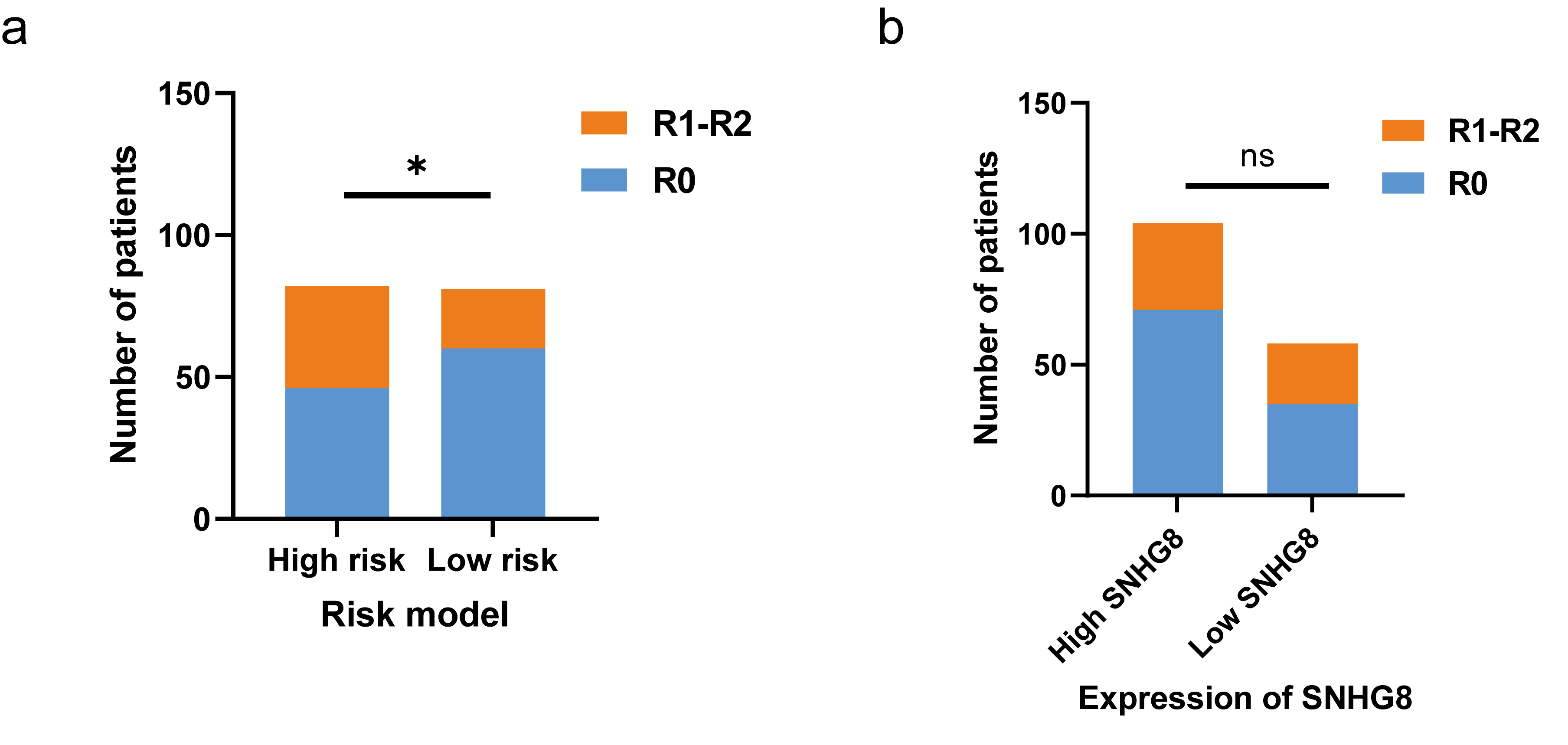

Supplement: Supplementary file 1 [file diagnostics-13-01697-s001.zip › supplementary files/figure S3.tif]

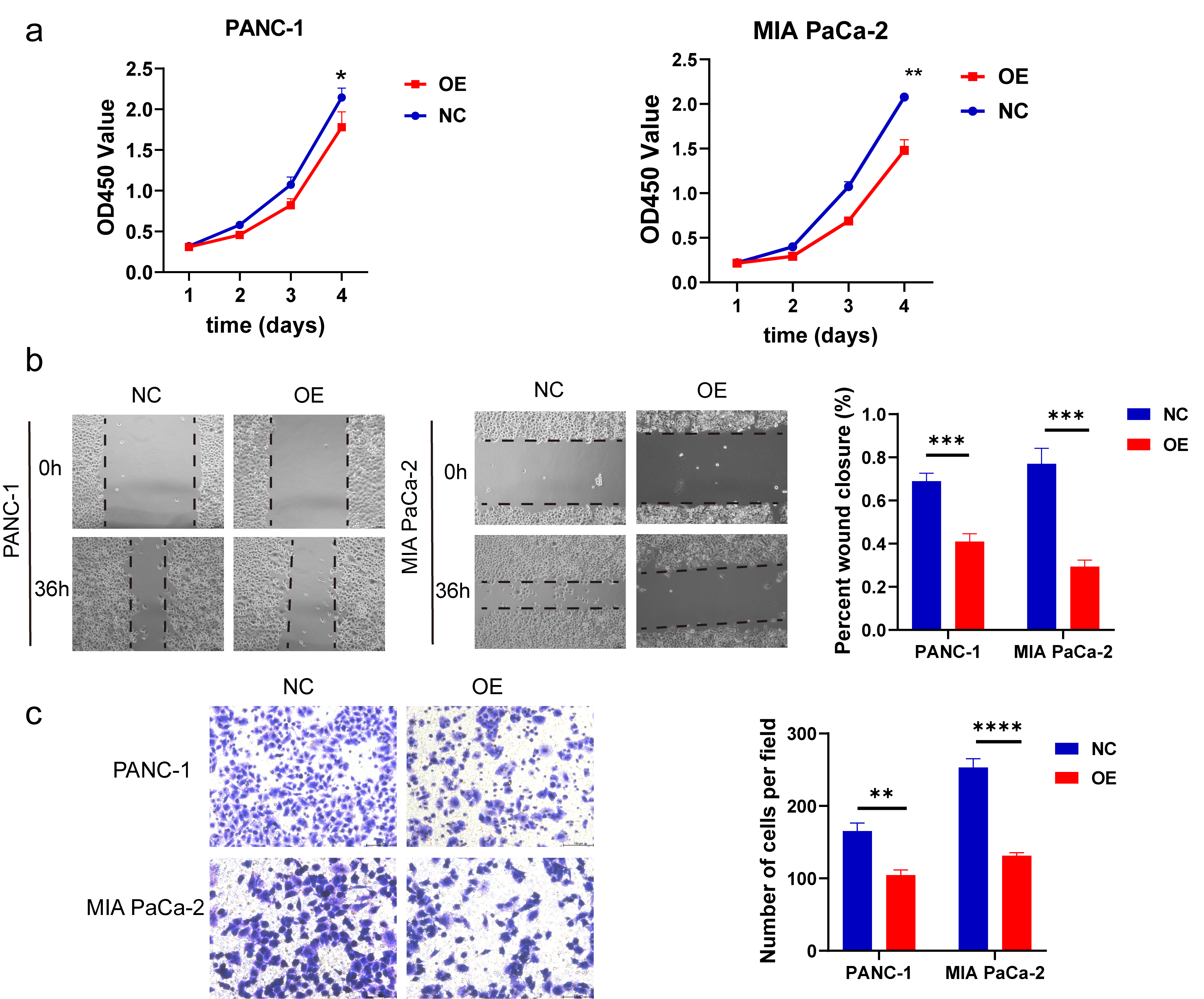

Supplement: Supplementary file 1 [file diagnostics-13-01697-s001.zip › supplementary files/Figure S4.tif]
